# The International Symposium on Avian Endocrinology, 1977–2024: Past, present and future

**DOI:** 10.1111/jne.13470

**Published:** 2024-11-21

**Authors:** John C. Wingfield

**Affiliations:** ^1^ Department of Neurobiology, Physiology and Behavior University of California Davis Davis California USA

**Keywords:** avian, endocrinology, neuroendocrinology, symposium

## Abstract

The First International Symposium on Avian Endocrinology (ISAE) was held 47 years ago at the Grand Hotel in Kolkata (Calcutta), India. Professor Asok Ghosh organized and convened the symposium, and Professor Donald S. Farner was President. The 1977 ISAE was convened at a time when neuroendocrine cascades were emerging as major pathways by which environmental events are perceived and transduced resulting in endocrine secretions that then orchestrate life history stages. Methods to measure hormone concentrations in blood and other tissues were relatively recent allowing the advance of laboratory and field investigations to explore ecological bases of endocrine control systems. The rise of evolutionary endocrinology and theory in ecological contexts followed—topics that are flourishing today. Studies on poultry continue to play central roles at ISAE meetings. In recent decades, the incorporation of genomics, transcriptomics, proteomics, epigenetics and other technologies provide us with an unprecedented array of tools to explore endocrinological processes at mechanistic levels we could never have dreamed of in 1977. The future looks to be an era of major advances in neuroendocrinology. What technologies will arise and transform our knowledge further? Artificial intelligence (AI) is emerging as a tool in avian endocrinology in at least research on endocrine disrupting chemicals. Will AI facilitate new advances and research directions across the field? The future of basic research has never been brighter than it is now. As in the past, ISAEs in the next decades will integrate new discoveries across environmental and applied biology. New challenges will doubtless appear.

## 1 INTRODUCTION

Almost 50 years ago, the First International Symposium on Avian Endocrinology (ISAE) was held in January 1977 at the Grand Hotel in Kolkata (Calcutta), India. Professor Asok Ghosh organized and convened the symposium, and Professor Donald S. Farner was President. Since then, the ISAE has been convened every 4 years except during the COVID‐19 pandemic when the Edinburgh ISAE was delayed by 2 years. The next ISAE meeting held 2 years later reset the long‐term schedule back to 4 years. The ISAE meeting held in Meerut in 2024 was convened by Professor Vinod Kumar, and Professor Simone L. Meddle served as President. They arranged a symposium that touched on many; the themes also addressed in 1977 include growth, neuroendocrine mechanisms of response to environment, and evolution of neuroendocrine/endocrine cascades. New levels of analysis using genomics, transcriptomics, and “omics” in general provided transformational insights into some “old” problems and generated hypotheses and predictions that paved the way to understanding novel concepts and related questions. We all look forward to the next ISAE in 2028 (venue to be determined) where exciting directions in neuroendocrinology will be addressed.

## 2 BRIEF HISTORY OF THE ISAE

**FIGURE 1:**
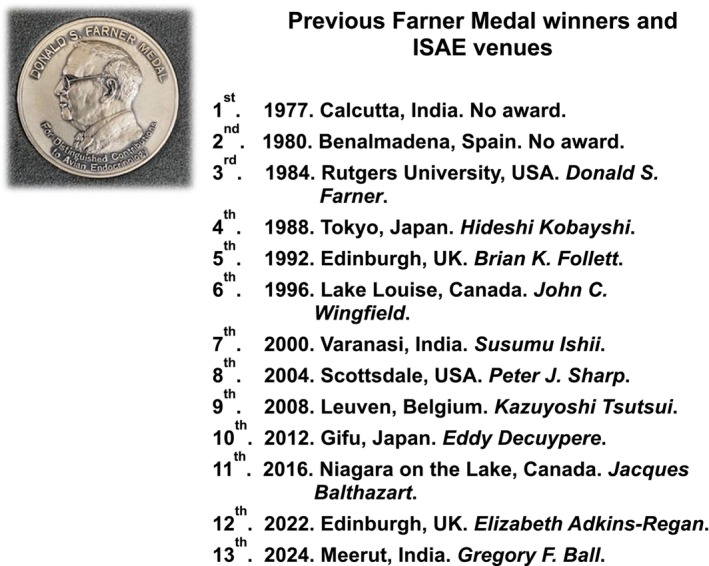
Venues of past International Symposia for Avian Endocrinology and awardees for the Donald S. Farner Medal for Avian Endocrinology.

Past venues of the ISAE are listed in Figure 1. At the third ISAE convened by Colin G. Scanes and held at Rutger's University in the USA, the Donald S. Farner Medal was inaugurated for distinguished contributions to avian endocrinology. The medal has been awarded at each symposium since then, and the awardees are also listed in Figure 1. These include Gregory F. Ball, the 2024 recipient.^1^

The 1977 ISAE came at a time when neuroendocrine cascades were implicated as critical components of pathways by which environmental events are perceived and transduced resulting in hormone secretions that then orchestrate the life cycle. Research presented in Kolkata was balanced among investigations on classic avian models such as the domestic fowl, and Japanese quail and continued to highlight work on wild species emerging as new models. In 1977, the now enormous field of endocrine disrupting chemicals was rising and continues to this day providing insights into more practical applications in endocrinology and the challenges presented by global change. The ability to measure hormone concentrations accurately in blood was still relatively recent in the 1970s but allowed the advent of fundamental investigations concerning secretion of hormones and whether they reflected activity of endocrine glands, target tissues, feedback, and so on. It soon became clear that although circulating levels of hormones provided useful information, they also indicated that mechanisms upstream and downstream of hormone secretions were critical and a source of diversity in mechanisms. These involved target cell sensitivity (e.g., receptors, hormone metabolizing enzymes), important feedback loops, and interactions with key autocrine and paracrine secretions. These in turn opened the way for laboratory and field investigations that explored ecological bases of endocrine control systems.^2^ Such new directions led to the rise of evolutionary endocrinology and theory, topics that are flourishing today, while agricultural studies on domestic fowl continue to play a central role integrating very different aspects of endocrinology in general—environment to molecules. ISAE meetings through the 1980s to the present incorporated the developing genomics, transcriptomics, proteomics technologies, and other fields so that today we have an unprecedented array of tools^2^ to explore endocrinological processes at mechanistic levels we could only have dreamed of in 1977.

## 3 THE NEXT SYMPOSIA

This brings us to the future—which lies with the younger generations of researchers and educators. The attendance in Meerut of undergraduate and graduate students, postdoctoral scholars, early career faculty, and their enthusiasm for the topics covered in ecological and evolutionary contexts provides a strong foundation for future ISAEs. Participation of researchers on domestic species will continue to enrich the proceedings with their insights and perspectives. Several questions arise: What technologies will develop, or at least appear on the horizon, that will transform our knowledge further? Is artificial intelligence emerging as a tool in avian endocrinology? The future of basic research has never been brighter than it is now, and looking forward, the ISAE will continue to be a forum for pioneering and integrative research directions. As pointed out at previous ISAEs, more attention should be paid to sex differences in environmental endocrinology to promote a complete and insightful picture.^3^ In recent years, it has become more and more apparent that sex differences in responsiveness to environmental cues are vital in assessing the impact of global change and conservation efforts in general.

The Meerut symposium was a spectacular array of papers using current molecular technologies, allowing comparisons across and within species and using new sophisticated computational capacities.^4^, ^5^ However, looking ahead to the progression of global change, will we be able to follow changes in transcriptomes, gene methylations, and proteomes over time by multiple sampling of tissues from within an individual? Access to enormous computation facilities will be critical for analysis of such huge data sets. In other words, what “big data” and computational facilities will be needed to interface with and assess other research directions, the evolution of neuroendocrine cascades and their actions at genomic and proteomic levels? As more and more avian genomes are sequenced, then these approaches at broad scales become entirely possible^6^ facilitated by new technologies for such massive integrative research. Will systems physiology provide a conceptual framework? Theoretical approaches including mathematical modeling will also be important to better understand current issues and identify new research directions of neuroendocrine cascades within ecological and evolution contexts. Mechanisms of gene–environment interaction perhaps assisted by artificial intelligence will provide, for example, insights for future research through interpretation of data^7^ and new insights into neural/neuroendocrine mechanisms.^5^

Other exciting technological advances include development of powerful tracking devices allowing us to follow even small birds over short periods of days, or eventually the lifetime.^8^ Integration with transcriptomics, etc., will provide truly unique data sets following animals in real time. The ISAE will be an ideal forum to highlight such ground‐breaking developments in the future.

It is important to mention that educational aspects of integration of new technologies applied to the organism in its environment will require students of the future to diversify their expertise and career objectives.^9^, ^10^ Teams of researchers proficient in a wide spectrum of the most recent technologies will become the driving forces of the future especially as the challenge of artificial intelligence looms ever larger. Avian neuroendocrinology will likely be a strong and vigorous community in the decades to come, and the ISAE will continue develop and feature advances at conceptual levels as well as focused studies in the laboratory and field. Birds in many ways are ideal study organisms, and several are already models—poultry, songbirds, seabirds, and others. They are generally abundant, tractable for observations as well as multiple captures in the field following timelines of phenology and switches in control systems.^10^

Future applications in global change, over a broad front, will encompass conservation, coping with impacts of invasive species, endocrine‐active pollutants, urbanization, and other anthropogenic activities. Avian endocrinology will provide a rich framework to assess and help to ensure future survival of life on Earth. The ISAE focus on mechanisms underlying environmental acclimation and adaptation was strong in 1977, has been sustained in the decades since, and will continue in the future.

## AUTHOR CONTRIBUTIONS

**John C. Wingfield:** Conceptualization; writing – original draft; writing – review and editing.

## CONFLICT OF INTEREST STATEMENT

The author declares no conflicts of interest.

## Data Availability

Data sharing is not applicable to this article as no new data were created or analyzed in this study.
